# Predictors of favorable outcome and mortality after endovascular thrombectomy in young Chinese patients with large vascular occlusions

**DOI:** 10.3389/fneur.2023.1227642

**Published:** 2023-07-12

**Authors:** Zhiqiang Li, Shuhui Wu, Fang Liang, Fengjiao Tan, Ning Li, Mengxin Bao

**Affiliations:** ^1^Department of Neurology, Liaocheng People’s Hospital, Liaocheng, Shandong, China; ^2^Department of Traditional Chinese Medicine, Liaocheng Third People’s Hospital, Liaocheng, Shandong, China; ^3^Department of Intensive Care Unit, Liaocheng Third People’s Hospital, Liaocheng, Shandong, China; ^4^Department of Neurology, Liaocheng Third People’s Hospital, Liaocheng, Shandong, China

**Keywords:** acute ischemic stroke, endovascular thrombectomy, large vessel occlusion, young stroke, functional outcomes

## Abstract

**Background:**

Endovascular thrombectomy (EVT) has evolved into the standard treatment for patients with acute ischemic stroke (AIS) and large vessel occlusion (LVO). However, little information is available on the management of EVT in young patients with AIS-LVO in China. The purpose of this study was to assess the favorable outcomes and mortality rates after 90 days of EVT in young Chinese patients with AIS-LVO and their predictors.

**Methods:**

This retrospective study included young Chinese patients aged 18–50 years with AIS-LVO. The primary efficacy endpoint was the modified Rankin scale (mRS) score at day 90, and the primary safety endpoint was mortality within 90 days. Using univariate and multivariate logistic regression analyses, the associations between clinical, imaging, and procedure variables and favorable (mRS 0–2) outcomes or mortality at 90 days were analyzed.

**Results:**

A total of 113 patients were included in the study with a mean age of 43.1 ± 6.3 years. Symptomatic intracranial hemorrhage (sICH) occurred in 8 (7.1%) patients. Favorable functional outcomes (mRS 0–2) were recovered in 42.5% of patients at 3 months. After 90 days, the mortality rate was 32.3%. Multivariate analysis revealed that the increase in admission NIHSS score was associated with a lower probability of functional independence (aOR 1.08, 95% CI 1.02–1.15, *p* = 0.01 and aOR 1.01, 95% CI 1–1.01, *p* = 0.008, respectively) and a higher probability of death at 90 days (aOR 1.1, 95% CI 1.03–1.18, *p* = 0.007 and aOR 1.00, 95% CI 1–1.01, *p* = 0.021, respectively).

**Conclusion:**

This study demonstrate that EVT provides higher rates of arterial recanalization, rather than better favorable outcomes and lower risk of death at 3 months in young Chinese patients with AIS-LVO. Increased NIHSS scores on admission may be associated with poor patient prognosis.

## Introduction

Stroke is the leading cause of adult disability in the United States (1), and stroke mortality rates in China are 343.4 per 100,000 person-years (2). The incidence of stroke in young patients comprises 15%–20% of the total, corresponding to approximately 30,000 strokes in young adults per year (3). Stroke in young people, particularly acute ischemic stroke (AIS) with large vessel occlusion (LVO), not only has long-term psychosocial, economic, and physical effects on patients but also has a devastating impact on the quality of life of patients and their caregivers (4). Younger patients with AIS-LVO have substantially different underlying stroke mechanisms than older adults, with fewer vascular risk factors and less pronounced structural vascular pathology changes (5–7). There are many causes of stroke in young people, of which cardiogenic embolism (20%–47%) and undetermined etiology are considered the more common causes (8–10). Due to the specificity of stroke in young people, the benefit of EVT for stroke in young people with LVO remains controversial. Previous studies have shown that younger LVO patients can derive more benefit from EVT than older patients (11, 12), with approximately 60%–66% of young LVO patients achieving good functional recovery at 3 months after EVT (6, 7). However, in the recent HERMES trial, the benefit of EVT in patients aged 18 to 49 years was not statistically significant for patients older than 50 years (13). The literature on acute EVT for AIS-LVO in young Chinese patients is limited. Therefore, this study aimed to assess the favorable outcomes and mortality rates after 90 days of EVT in young Chinese patients with AIS-LVO and their predictors.

## Methods

### Patient selection and data collection

In this study, we retrospectively examined all patients with anterior circulation who received EVT within 6 h of symptom onset and patients with posterior circulation AIS-LVO who received EVT within 24 h at Liaocheng Brain Hospital in Shandong Province between January 2017 and February 2023. A total of 1,384 patients met these criteria, with a total of 135 patients aged 18–49 years, 17 of whom underwent only arterial thrombolysis. Four other patients had *in situ* stenosis and received emergency stenting or balloon dilation, and 1 patient had emergency venous sinus thrombectomy. After patients with nonemergency thrombectomy were excluded, we had 113 young Chinese patients with AIS-LVO who were finally included in this study.

### Demographic data and comorbid risk factors

Baseline patient characteristics, including sex, age, and the National Institutes of Health Stroke Scale (NIHSS) score, reflected stroke severity on admission (14). Comorbid cardiovascular risk factors (CVRFs) mainly included hypertension, diabetes, atrial fibrillation, stroke, and smoking.

### Stroke etiology

Stroke etiology was assessed according to the Trial of ORG 10172 in the Acute Stroke Treatment (TOAST) classification system (15). Furthermore, considering the specificity of LVO in young people, we also referred to some previous criteria (16). We finally classified the etiology into the categories of cardiogenic, atherosclerotic, arterial dissection, unexplained, and other causes.

### Clinical variables and imaging variables

All patients were screened by computed tomography (CTA), magnetic resonance angiography (MRA), or carotid ultrasound, and the diagnosis of acute LVO was confirmed by digital subtraction angiography (DSA). The sites of LVO included the anterior circulation, which includes the internal carotid artery (ICA), the terminal ICA (T-lesion; T occlusion), the first and second segments of the middle cerebral artery (MCA M1 M2), and the posterior circulation, which includes the basilar artery (BA) and vertebral artery (VA). Other clinical variables included whether intravenous thrombolysis was performed, the type of drug used for intravenous thrombolysis, symptoms to admission, symptoms to puncture, and symptoms to recanalization.

### EVT techniques and procedure variables

The vast majority of our included patients underwent EVT procedures under general anesthesia with transoral tracheal intubation. The procedure included 5F or 6F intermediate catheter aspiration thrombectomy, aspiration combined with stent retriever thrombectomy, or stent retriever thrombectomy alone. Successful recanalization was defined as a modified thrombolysis score in cerebral infarction (mTICI) score of 2b or 3 (17).

### Outcome measures

The primary outcome was the score on the modified Rankin scale (mRS) on day 90, and mortality within 90 days. The mRS was rated blindly at two follow-ups 90 days after EVT, either via telephone interviews with the patients and their families or during the hospital visit. A favorable functional outcome was defined as mRS 0–2 at 90 days after treatment. Symptomatic intracerebral hemorrhage (sICH) was defined as any hemorrhage accompanied by a worsening of the NIHSS score of ≥4 points within 48 h after EVT. Intracerebral hemorrhages were evaluated on the Heidelberg Bleed Classification (18). NIHSS at 24 h after EVT was also collected.

### Statistical analysis

Data are presented as the mean ± standard deviation (SD) or median (interquartile range) for continuous variables and as frequency or percentage for categorical variables. For the analysis of baseline characteristics, significant differences were tested with a t test or one-way ANOVA for continuous variables and a chi-square or Fisher test for categorical variables. Odds ratios with 95% confidence intervals were computed using univariate and multivariate logistic regression models with admission NIHSS score as dependent variables. All analyses were performed using R Statistical Software (http://www.R-project.org, The R Foundation) and the Free Statistics analysis platform (Beijing, China).

## Result

During the study period, 113 patients aged 18–49 years were enrolled in the study. The average age of these patients was 43.1 ± 6.3 years, and 21 (18.6%) of them were female. The result of the NIHSS score at 24 h postoperatively was 15.2 ± 8.1, which had a significant improvement compared to its NIHSS scores were 19.6 ± 9.0 at the time of admission (*p* < 0.05). Approximately 60.2% of young stroke patients had a smoking habit. Hypertension was the most prevalent comorbidity (47.8%), followed by diabetes mellitus (19.5%), while only 10.6% of patients were confirmed to have atrial fibrillation before hospitalization. Based on imaging characteristics, approximately 80.5% of the lesions were in the anterior circulation, especially in the M1 segment of the middle cerebral artery (42.5%). Approximately 42.4% of the patients received bridge therapy, and the most commonly used intravenous thrombolytic drug was rt-PA.

The median times from symptoms to admission, puncture and recanalization was 4 h, 4.3 h, and 6.9 h, respectively. The success rate of the procedure (mTICI 2b–3) was 88.5%. Among the cases included in the study, 18 (27.4%) patients presented with ICH, of which 10 (18.6%) were sICH. At 3 months, 42.5% (48/113) of the patients had recovered a favorable functional outcome (mRs 0–2). The mortality rate was 32.3% (21/113) after 90 days. Etiologically, the most common stroke in young Chinese was LAA (38.1%), followed by CE (23.9%). Approximately 64.6% (73/113) of patients developed complications in the perioperative period, the most common of which was pulmonary infection (33.6%). The characteristics of the baseline are detailed in Table 1.

**Table 1:** Baseline data characteristics of young Chinese stroke patients.

Variables	Total (n = 113)	mRS ≤2 (n = 48)	mRS >3 (n = 65)	*p*-value
*Demographics*				
Gender, female, *n* (%)	21 (18.6)	9 (18.8)	12 (18.5)	0.969
Age (year), Mean ± SD	43.1 ± 6.3	42.8 ± 7.0	43.4 ± 5.8	0.604
NIHSS score at admission, Mean ± SD	19.6 ± 9.0	17.2 ± 8.7	21.4 ± 8.9	0.012
*Medical history*				
Smoking, *n* (%)	68 (60.2)	32 (66.7)	36 (55.4)	0.226
Hypertension, *n* (%)	54 (47.8)	19 (39.6)	35 (53.8)	0.134
Diabetes, *n* (%)	22 (19.5)	10 (20.8)	12 (18.5)	0.753
Atrial fibrillation, *n* (%)	12 (10.6)	3 (6.2)	9 (13.8)	0.195
Stroke, *n* (%)	4 (3.5)	3 (6.2)	1 (1.5)	0.31
Cardiac valve disease, *n* (%)	6 (5.3)	2 (4.2)	4 (6.2)	1
Others, *n* (%)	23 (20.4)	8 (16.7)	15 (23.1)	0.403
*Imaging features*				
Lesion location, *n* (%)				0.083
ICA	18 (15.9)	4 (8.3)	14 (21.5)	
ICA-T	12 (10.6)	3 (6.2)	9 (13.8)	
M1	48 (42.5)	25 (52.1)	23 (35.4)	
M2	10 (8.8)	7 (14.6)	3 (4.6)	
VA	3 (2.7)	1 (2.1)	2 (3.1)	
BA	22 (19.5)	8 (16.7)	14 (21.5)	
*Treatment details*				
IVT, *n* (%)				0.425
rtPA	37 (32.7)	13 (27.1)	24 (36.9)	
Urokinase	11 (9.7)	4 (8.3)	7 (10.8)	
Symptoms to admission (hour), median (IQR)	4.0 (3.0, 6.0)	4.0 (3.0, 6.0)	4.0 (3.0, 6.0)	0.428
Symptoms to puncture (hour), median (IQR)	4.3 (3.2, 6.2)	4.8 (3.2, 6.4)	4.2 (3.2, 6.2)	0.519
Symptoms to recanalization (hour), median (IQR)	6.9 (5.3, 9.0)	6.8 (5.3, 8.7)	6.9 (5.8, 9.1)	0.682
*Technical efficacy*				
TICI, *n* (%)				0.755
0–2a	13 (11.5)	5 (10.4)	8 (12.3)	
2b–3	100 (88.5)	43 (89.6)	57 (87.7)	
*Outcome*				
NIHSS at 24 h after EVT, mean ± SD	15.2 ± 8.1	12.6 ± 7.5	17.1 ± 8.1	0.003
ICH, *n* (%)				0.149
sICH	8 (7.1)	1 (2.1)	7 (10.8)	
aICH	10 (8.8)	3 (6.2)	7 (10.8)	
Death within 3 months, *n* (%)	21 (18.6)	0 (0)	21 (32.3)	<0.001
*Possible etiology*				
Possible etiology, *n* (%)				
Dissection	12 (10.6)	5 (10.4)	7 (10.8)	0.952
CE	27 (23.9)	11 (22.9)	16 (24.6)	0.834
LAA	43 (38.1)	18 (37.5)	25 (38.5)	0.917
Unknown	27 (23.9)	12 (25)	15 (23.1)	0.813
Other reasons	4 (3.5)	2 (4.2)	2 (3.1)	1
*Perioperative complications*				
Pneumonia, *n* (%)	38 (33.6)	14 (29.2)	24 (36.9)	0.388
Large cerebral infarction, *n* (%)	12 (10.6)	4 (8.3)	8 (12.3)	0.498
Cerebral hernia, *n* (%)	8 (7.1)	1 (2.1)	7 (10.8)	0.135
Gastrointestinal hemorrhage, *n* (%)	3 (2.7)	1 (2.1)	2 (3.1)	1
Epilepsy, *n* (%)	5 (4.5)	0 (0)	5 (7.7)	0.073
Others, *n* (%)	7 (6.5)	4 (8.7)	3 (4.9)	0.46

LAA; large artery atherosclerosis, CE; cardioembolism, sICH; symptomatic intracranial hemorrhage, aICH; asymptomatic intracranial hemorrhage, TICI; thrombolysis in cerebral infarction, IVT; intravenous thrombolytic therapy, VA; vertebral artery, BA; basilar artery, ICA; internal carotid arteries, ICA-T; terminal T-occlusion of the internal carotid artery.

The following variables were identified as univariate predictors of a favorable functional outcome at 3 months: NIHSS score at admittance (OR 1.06, 95% CI 1.01–111; *p* = 0.015). According to multivariate analysis, NIHSS score at admission (aOR 1.01, 95% CI 1–1.01, *p* = 0.008) was an independent inversed predictor of a favorable functional outcome at 3 months. The univariate and multifactorial analyses of predictors of a favorable functional outcome are detailed in Table 2.

**Table 2:** Univariate and multivariate analyses of predictors of favorable functional outcome at 3 months in Chinese young stroke patients receiving EVT.

Variable	Univariate analysis		Multivariate analysis	
	OR (95% CI)	*p*-value	OR (95% CI)	*p*-value
Gender	1.02 (0.39–2.66)	0.969		
Age (year)	1.02 (0.96–1.08)	0.6		
NIHSS score at admission	1.06 (1.01–1.11)	0.015	1.08 (1.02–1.15)	0.01
Smoking	0.62 (0.29–1.35)	0.227		
Hypertension	1.33 (0.63–2.81)	0.461		
Diabetes	0.86 (0.34–2.2)	0.753		
Atrial fibrillation	1.54 (0.44–5.46)	0.5		
Stroke	0.23 (0.02–2.33)	0.215		
Cardiac valve disease	3.92 (0.44–34.67)	0.22		
Lesion location^b^	0.73 (0.28–1.91)	0.519		
IVT (rt-PA)	1.68 (0.73–3.87)	0.22		
IVT (Urokinase)	1.6 (0.43–5.98)	0.488		
Symptoms to recanalization (hour)	1 (0.92–1.1)	0.926		
TICI^a^	0.83 (0.25–2.71)	0.756		
sICH	5.67 (0.67–47.75)	0.11		
Arterial dissection	1.04 (0.31–3.49)	0.952		
Cardiogenic	1.1 (0.46–2.64)	0.834		
Atherosclerosis of large arteries	1.04 (0.48–2.25)	0.917		
Other causes type	0.73 (0.1–5.38)	0.758		

TICI; thrombolysis in cerebral infarction, IVT; intravenous thrombolytic therapy, sICH; symptomatic intracranial hemorrhage.

a 0–2a vs. 2b–3.

b VA vs. ICA.

The following variables were identified as univariate predictors of mortality at 90 days: NIHSS score at admission (OR 1.1, 95% CI 1.04–1.17; *p* = 0.001), hypertension (OR 3.4, 95% CI 1.21–9.55; *p* = 0.02), and location of the injury (OR 0.22, 95% CI 0.08–0.62; *p* = 0.004). Multivariate analysis revealed that the NIHSS score at admission (aOR 1.1, 95% CI 1.03–1.18, *p* = 0.007) was independent predictors of mortality at 90 days. The univariate and multifactorial analyses of predictors of mortality at 90 days are detailed in Table 3.

**Table 3:** Univariate and multivariate analysis of mortality factors at 3 months in Chinese young stroke patients receiving EVT.

Variable	Univariate analysis		Multivariate analysis	
	OR (95% CI)	*p*-value	OR (95% CI)	*p*-value
Gender	0.67 (0.22–2.11)	0.497		
Age (year)	1.04 (0.95–1.12)	0.396		
NIHSS score at admission	1.1 (1.04–1.17)	0.001	1.1 (1.03–1.18)	0.007
Smoking	0.53 (0.21–1.39)	0.197		
Hypertension	3.4 (1.21–9.55)	0.02	2.97 (0.95–9.26)	0.061
Diabetes	1.38 (0.44–4.28)	0.579		
Atrial fibrillation	0.37 (0.04–3.02)	0.352		
Stroke	1.48 (0.15–15.01)	0.738		
Cardiac valve disease	0.87 (0.1–7.86)	0.901		
Lesion location^b^	0.22 (0.08–0.62)	0.004	0.21 (0.04–1.1)	0.064
IVT (rt-PA)	1.28 (0.44–3.72)	0.646		
IVT (Urokinase)	3.14 (0.77–12.76)	0.109		
Symptoms to recanalization (hour)	1.09 (0.98–1.2)	0.115		
TICI^a^	3 (0.37–24.45)	0.305		
Arterial dissection	0.86 (0.17–4.27)	0.857		
Cardiogenic	1.8 (0.64–5.06)	0.265		
Atherosclerosis of large arteries	0.59 (0.21–1.67)	0.325		
Unknown cause	0.99 (0.33–3.03)	0.992		

TICI; thrombolysis in cerebral infarction, IVT; intravenous thrombolytic therapy.

a 0–2a vs. 2b–3.

b VA vs. ICA.

## Discussion

EVT has become a standard procedure for acute LVO, but data on LVO in Chinese youth are rare. To our knowledge, this is the first study to examine the favorable outcomes and mortality rates after 90 days of EVT in young Chinese patients aged 18 to 50 years and its impact on their clinical prognosis and risk of mortality. Although different from other studies in terms of etiology, a favorable functional prognosis in EVT can be achieved in nearly half of young Chinese AIS patients.

The definition of youth varies in different studies, with age ranges of 18–40 years, 18–49 years, or 18–55 years (6, 19, 20). In studies of young people with stroke combined with LVO for EVT, the age range of 18–49 years was mainly chosen. The mean age of the patients in our study was 43.1 ± 6.3 years, which is higher than the 41.4 years in previous studies (7). Unlike elderly individuals, young people have a much lower incidence. In the MR CLEAN Registry, a prospective survey from the Netherlands, approximately 10% of patients treated with EVT were young patients with LVO (6), which is in line with our data of 8.2% (113/1384). The same results have been reported in other studies, where 5.5%–10% of patients are 18 to 49 years old (6, 13, 21). Our findings suggest that the NIHSS score at admission was 19.6 ± 9.0, markedly different from previous studies in which young patients had a lower baseline NIHSS score of 14, while even in older adults, the NIHSS score was only 16, although it was only included in the anterior circulation study (6). In another study on real-world multicenter experience, young people had a median of 13, and older people had a median of only 15 (7). The high NIHSS scores at admission were due to the large period of the included studies, some of which included posterior circulation strokes with high NIHSS scores.

Unlike NIHSS, the rate of successful recanalization (TICI ≥2b) was 88.5% (100/113), which is consistent with previous studies, 64.2%–88.5% (6, 7, 22). All of these studies suggest that the success rates are higher in young stroke patients, possibly related to potentially less tortuous arterial anatomy and atherosclerosis in younger patients (23). In our study, 57.5% (65/113) of the patients were treated with direct embolization without a bridge. Several factors influence this, such as time of admission, contraindications to thrombolysis, and patient family wishes, which are particularly common in young stroke patients with AIS-LVO. Controversy persists regarding bridging therapy and direct arterial thrombolysis, for example, in studies such as the DIRECT MT (24) and DEVT (25). The same issues exist in young Chinese AIS-LVO patients.

Our study showed that the proportion of young Chinese LVO patients who achieved a favorable functional outcome at 3 months after EVT was 42.5% (48/113), which is lower than the 61% in previous studies (6), although it was mainly about anterior circulation LVO. Our study’s findings were in line with a meta-analysis on posterior circulation EVT, in which the rate of favorable functional outcome was 45.1% (26), but similar to those on anterior circulation, which revealed that 56% of patients aged 18 to 49 had a favorable outcome (13). In our study, an increase in the NIHSS score at admission was associated with a lower probability of functional independence at 90 days. Our results are consistent with a previous literature, in which ASPECTS and NIHSS were independent predictors of a favorable outcome (27)，but different from the other, in which shorter time from onset to arrival and reperfusion, successful recanalization and absence of hemorrhagic transformation are the predictors of favorable clinical outcome (28). Furthermore, 19.5% (22/113) of the patients in our study had posterior circulation involvement, which prevented us from applying the ASPECT score to more specifically assess the patient’s condition at admission in terms of imaging.

The safety outcomes of our study were mortality at 3 months and sICH. The mortality rate was 32.3% (21/113) in our study due to the inclusion of patients with higher NIHSS scores on admission. The mortality rate in our study is much higher than that in a previous study that showed a mortality of 7% in youth (6). Another paper on EVT in young adults mentioned that the 90-day overall mortality rate was 12%, with predictors including time from onset to arrival and reperfusion as well as duration of the procedure (28). In our study, 18 (27.4%) patients presented with intracranial hemorrhage, of which 10 (18.6%) were sICH, which is higher than the previous study 2.8%–6.5% (6, 7, 22). We consider the reason for the higher incidence of sICH in our study to be related to the higher admission score of the NIHSS and the higher recanalization rate.

The etiology of stroke in young people is complex, and Western studies have shown that the common causes of stroke in young people are mostly nonatherosclerotic. Etiologically, the main cause of stroke in young people in the West is of unknown etiology or cardiogenic emboli. Previous literature reported 25%–50% of cryptogenic strokes in young people, while cardiogenic emboli accounted for 25% and vascular dissection for 20% (20). In contrast, the main cause of stroke in the Asian youth population is atherosclerosis. For example, in Mingyu Tang et al. they showed that the most common subtype was atherosclerosis-related risk factors (61.7%), especially in patients aged >35 years and in males (29). It is worth noting that these studies included young patients with stroke overall and not only patients with an LVO. However, our study revealed that the most common etiology of stroke in young adults in China was LAA (38.1%), followed by cardioembolism (23.9%) and unexplained stroke (23.9%). This is similar to another posterior circulation study in China, which showed etiologies in young patients, including LAA (67.11%), cardioembolism (15.13%), and vessel dissection (5.26%) (30). Other studies have shown that cervical dissection and nonarrhythmic heart disorders are frequent causes of stroke in young patients (12, 23, 31). This discrepancy was also present in our article. We consider that the reasons for this discrepancy may exist in population characteristics such as the presence of intracranial atherosclerosis in Asian populations and beginning of the internal carotid artery in Western populations (32), as well as lifestyle habits such as high salt, high fat, frying and stir-fry cooking styles and smoking and alcohol habits.

There are still some limitations to this study. First, this study is a single-center, small-sample, nonrandomized controlled study, and its inherent bias could not be removed. Second, the ASPECT score at admission was not included because the ASPECT score does not combine the anterior and posterior circulations and cannot be applied to score cases of cerebral infarction involving the combined anterior and posterior circulation. Furthermore, the period was long, not only in the middle of the renewal of the aspiration catheter but also in the absence of CTP examination in the earlier period. In addition, there is some young stroke etiology onset that needs time to be validated. These factors should be considered in future studies. Finally, the collateral status may affect the prognosis, and we were not able to collect relevant data from our center.

## Conclusion

The results of this study demonstrate that atherosclerotic factors remain the main etiology of AIS-LVO in young patients in China. EVT provides remarkably high rates of arterial recanalization rather than better favorable outcomes and lower risk of death at 3 months in young Chinese patients with AIS and LVO. Increased NIHSS scores on admission may be associated with poor patient prognosis. Larger, multicenter, randomized controlled trials are needed to confirm the results of this study.

## Data availability statement

The raw data supporting the conclusions of this article will be made available by the authors, without undue reservation.

## Ethics statement

The studies involving human participants were reviewed and approved by Liaocheng People's Hospital. The patients/participants provided their written informed consent to participate in this study.

## Author contributions

ZL and NL executed the procedure. ZL, MB, and SW wrote the manuscript. FL and FT contributed to the table descriptions. All authors contributed to the article and approved the submitted version.

## Conflict of interest

The authors declare that the research was conducted in the absence of any commercial or financial relationships that could be construed as a potential conflict of interest.

## Publisher’s note

All claims expressed in this article are solely those of the authors and do not necessarily represent those of their affiliated organizations, or those of the publisher, the editors and the reviewers. Any product that may be evaluated in this article, or claim that may be made by its manufacturer, is not guaranteed or endorsed by the publisher.

## Abbreviations

EVT: endovascular thrombectomy
AIS: acute ischemic stroke
LVO: large vessel occlusion
sICH: symptomatic intracranial hemorrhage
NIHSS: National Institutes of Health Stroke Scale
DSA: digital subtraction angiography
